# Serotonin transporter polymorphism (5-HTTLPR) is associated with post-awakening cortisol measures in previous depression

**DOI:** 10.1016/j.cpnec.2026.100363

**Published:** 2026-07-22

**Authors:** R. Jonassen, Ø. Øverli, E. Hilland, H.L. Nilsen, Q.Y. Esbensen, L. Lirussi, H. Heiberg, K.H. Rønning, M. Gorissen, B. Kraft, R. Bø, C.J. Harmer, N.I. Landrø

**Affiliations:** a Oslo Metropolitan University, Department of Health Sciences, Oslo, Norway; b Norwegian University of Life Sciences, Department of Animal and Aquacultural Sciences, Ås, Norway; c Clinical Neuroscience Research Group, Department of Psychology, Oslo, Norway; d Diakonhjemmet Hospital, Division of Psychiatry, Oslo, Norway; e Department of Microbiology, Oslo University Hospital, Oslo, Norway; f Division of Medicine, Akershus University Hospital, Akershus, Norway; g Department of Biosciences, University of Oslo, Oslo, Norway; h Department of Plant and Animal Biology, Radboud Institute for Biological and Environmental Sciences, Radboud University, Nijmegen, the Netherlands; i Psychopharmacology and Emotion Research Laboratory, University of Oxford, Oxford, United Kingdom

**Keywords:** Cortisol awakening response, 5-HTTLPR, Major depressive disorder, Salivary cortisol, Area under the curve, HPA axis

## Abstract

Elevated cortisol levels have been linked to major depressive disorder (MDD), but cortisol dynamics vary substantially across individuals and clinical states. Genetic variation in the serotonin transporter polymorphism (5-HTTLPR) has been proposed as one contributor to differences in hypothalamic-pituitary-adrenal axis functioning. The present study examined associations between cortisol awakening measures and 5-HTTLPR genotype groups in individuals with a history of depression compared with healthy controls, and explored whether current depressive symptom severity was related to cortisol indices. A total of 108 individuals with a history of depression were genotyped and compared with 59 healthy controls. Participants completed structured diagnostic interviews and self-reported depressive symptoms. Salivary cortisol was sampled in the evening and at five consecutive timepoints after awakening (15-min intervals). Cortisol was analyzed using a linear mixed-effects model of repeated measures and by cortisol awakening response (CAR) summary indices: area under the curve with respect to ground (AUCg) and area under the curve with respect to increase (AUCi). Cortisol changed significantly over time, but there was no evidence that genotype groups differed in the overall cortisol timecourse. In CAR summary analyses, group differences were observed for AUCg, whereas AUCi did not show corresponding group differences. Regression analyses indicated that BDI-II was not associated with AUCg or AUCi after adjustment for covariates. These findings suggest that 5-HTTLPR-related differences, when present, may be more detectable in overall post-awakening cortisol output than in baseline-relative increase.

## Highlights

• Cortisol changed over time, but genotype groups did not differ in trajectory.
• AUCg showed a group effect with a dose-related gradient by short-allele count.
• AUCi did not show corresponding group differences.
• BDI-II was not associated with AUCg or AUCi after covariate adjustment.

## 1 Introduction

A history of depression substantially increases the likelihood of subsequent depressive episodes, and risk tends to increase with each additional episode [1,2]. Persistent symptoms after recovery are also common and are frequently discussed as “residual symptoms,” particularly among individuals with recurrent depression [3,4] (see Table 1).

Cortisol and serotonin are central components of the biological stress response and emotional regulation, and dysregulation in these systems has been linked to depressive states and depression vulnerability [5,6]. The hypothalamic–pituitary–adrenal (HPA) axis is the primary endocrine system supporting adaptation to internal and external stressors, with glucocorticoids (cortisol in humans) providing key feedback regulation through receptors in the central nervous system. In depression, altered HPA-axis functioning has been reported, including elevated cortisol levels and disturbances in diurnal rhythmicity in some samples, although findings are heterogeneous across studies and clinical presentations [[7], [8], [9]]. Elevated cortisol has also been reported in individuals with a history of depression compared with healthy controls [9,10]. The cortisol awakening response (CAR), typically assessed as the early post-awakening rise in cortisol, is a distinct component of diurnal cortisol dynamics and has been associated with stress exposure and stress-related processes [11]. CAR patterns have been linked to psychosocial factors such as anticipation of the upcoming day and cognitive-emotional processes, but their interpretation in relation to psychopathology remains complex [12,13].

Serotonin is also implicated in neurodevelopment and emotion-related circuitry and may influence vulnerability to psychopathology through effects on synaptic plasticity and neural network function [5]. The serotonin transporter-linked polymorphic region (5-HTTLPR) in the promoter of SLC6A4 is commonly described in terms of short and long alleles [14], and has been linked to individual differences in emotion processing and stress-related biology [15]. A meta-analysis reported that 5-HTTLPR variation is associated with HPA-axis reactivity to acute psychosocial stress, with short-allele carriers showing higher cortisol responses than long-allele carriers [16]. Prior work also suggests that the association between genotype and cortisol-related outcomes may be more pronounced in the context of stressful life experiences [17,18].

Importantly, both acute and chronic stress can influence cortisol dynamics, potentially in different ways; acute stress is typically characterized by a transient cortisol increase followed by recovery, whereas chronic stress and allostatic load may contribute to longer-term alterations in baseline levels and diurnal patterning [19].

Against this background, the present study aimed to clarify associations between depression history, 5-HTTLPR genotype groups, and morning cortisol dynamics. To align with current CAR methodology and to strengthen interpretability, cortisol was analyzed using linear mixed-effects modeling of repeated measures and by CAR summary indices computed across the post-awakening window, distinguishing overall cortisol output (AUCg) from baseline-relative increase (AUCi). We expected cortisol to show systematic change across the morning sampling window and tested whether genotype groups within the depression-history sample differed in cortisol measures when compared with healthy controls. Finally, we explored whether current depressive symptom severity (BDI-II) was associated with AUC-based cortisol indices.

## 2 Materials and methods

### 2.1 Participants and recruitment procedures

An MDD sample and healthy controls were invited to a clinical trial and an experimental study at the Department of Psychology, University of Oslo, Norway, between 2015 and 2018. This study was exploratory and represents secondary use of baseline data drawn from two pre-registered studies [20,21]. However, the present analyses were not themselves pre-registered. Patients were recruited from three clinical sites and social media platforms, and outreach for healthy controls was conducted through local advertisements and social media platforms. Candidates were pre-screened by phone following the exclusion criteria before in-person formal clinical evaluation took place. Individuals diagnosed with current depression, current or former neurological disorders, psychosis, bipolar spectrum disorders, substance use disorders, attention deficit disorder, or head trauma were excluded. Advancements in technology in 2023 allowed for gene sequencing and genotyping to be conducted on stored extracted DNA. All participants who provided combined cortisol samples and DNA and who did not meet any exclusion criteria were defined as eligible for participation.

### 2.2 Clinical screening and symptom assessment

We used Mini International Neuropsychiatric Interview (MINI) 6.0, a structured diagnostic interview, to assess the presence, history, and severity of a wide range of mental health disorders. MINI 6.0 covers major psychiatric disorders such as depression, anxiety, bipolar disorder, schizophrenia, and substance use disorders. The Beck Depression Inventory (BDI-II), which consists of 21 items, was used as a measure of the severity of depression symptoms [22].

### 2.3 Cortisol sampling and radioimmunoassay

Saliva samples were obtained using the Sarstedt Cortisol Salivette® Device, which uses polypropylene/low-density polyethylene tubes with a separate internal detachable compartment containing a cotton swab. Awakening time and sampling times were self-reported (no actigraphy/sleep-tracking and no electronic monitoring, time-stamped photos/codes, or text-message verification). Adherence was supported by providing sampling kits in an envelope with written instructions on the outside describing the sampling schedule and procedures, reinforced by standardized oral instruction and Q&A from laboratory staff during the diagnostic screening visit on the same day as the first (evening sample). Because objective timing verification was unavailable, we could not exclude samples based on verified timing error (e.g., Δt = 0 ± 5 min) or incorporate verified deviations into estimation models. Participants received guidance intended to minimize acute behavioral influences during morning sampling (e.g., avoid food/drinks other than water, smoking, and strenuous exercise), and were instructed to postpone sampling under atypical conditions (e.g., acute illness, jet lag, shift work); relevant medical exclusions were applied per protocol where applicable. Sex and age were assessed and controlled statistically, whereas state influences such as weekday/weekend, psychosocial anticipation, and verified awakening time were not reported. Before use, the Salivette containers were stored dry at room temperature; after use, the participants were instructed to store the containers in a freezer. Sample T1 was taken in the evening of day one, and samples T2–6 were taken when the participants woke up the next morning. Participants were instructed to take evening samples between 8 p.m. and 10 p.m. and morning samples between 7 a.m. and 9 a.m., as soon as they woke up, and a new sample every 15 min (5 time points). Participants were asked to schedule extra time (approximately 1 h) for taking morning samples. Samples were delivered by the patients themselves to the Department of Psychology and stored at −18**°**C, until samples were transferred to −80**°**C within two weeks. The samples were thawed on ice and cold centrifuged at 4**°**C, 1000 * rcf for 15 min. The saliva samples were then transferred into 1.5 ml Eppendorf tubes and stored at −80**°**C. The samples were brought to Radboud University in Nijmegen on dry ice for radio immunoassay (RIA) analysis. The protocol used for cortisol RIA in micro plates was refined from a previous methodology, as described by Ref. [23]. In short, 3–5 96-well microassay plates (Greiner Bio-One: 655094; White/μClear - high-binding) were prepared each day. Wells were prepared by adding cortisol antibody (Abcam: ab1949; Cortisol Antibody [xm210] monoclonal and IgG purified) diluted in coating buffer to all wells, except A-specifics, which received coating buffer only. The plates were incubated overnight at 4°C. Following incubation, the wells were washed with 200 μl wash buffer and then with 200 μl block buffer. The plates were then incubated in a heat cabinet at 37°C for 1 h. Blocking buffer was removed from the wells by decanting. Immediately thereafter, 10 μl standards (Sigma: H4001-5G; hydrocortisone ≥98% HPLC) and saliva samples (thawed on ice for ∼1–2 h) were added in duplicates to designated wells. Finally, 90 μl 3H-cortisol tracer (PerkinElmer: #NET396250UC - Hydrocortisone (Corstiol,[1,2,6,7-3H(N)]-),[1,2,6,7-3H(N)]- 250 μCi (9.25MBq) was added into each well and left to cold incubate overnight at 4°C. Thereafter, the incubation plates were washed three times with wash buffer. Prior to β-measurement, scintillation solution was added to all wells. The values obtained were directly translated into saliva cortisol concentrations. The samples were assayed in duplicate (*r* = .8849). The intra-assay coefficients of variation for our low- and high-quality control standards were 4.3% and 6.7%, respectively. The inter- and intra-assay variation coefficients were 12.5% and 2.5%, respectively. The cross-reactivity of the antibody with cortisone was <1%.

### 2.4 DNA extraction, purification, and polymerase chain reaction (PCR)

To obtain DNA from buccal epithelium cells, the study participants were instructed to rub an Isohelix SK-1S DNA Buccal Swab for 1 min on the inner cheek. The DNA samples were stored at room temperature until analysis. DNA isolation was carried out using a DNeasy Blood and Tissue kit from Qiagen or a BFK-50 kit from Isohelix. The following procedure description is freely available for the BFK-50 Isohelix kit. In short, 20 μl PK solution was added to the tube containing the buccal swab. After a 30 min incubation, the entire sample was transferred to a 1.5 ml tube, and 400 μl BP solution was added. The samples were then centrifuged at maximum speed (13.4K *rpm*/12,000 × *g*) for 10 min. The resulting pellet contained both the DNA and impurities. The supernatant was carefully removed, after which 50–150 μl TE solution was added to the pellet, and the tube was vortexed to resuspend the pellet in the solution. After 2–5 min, the DNA was fully hydrated. To remove the undissolved impurities, the tube was spun for another 15 min at maximum speed. Finally, the supernatant containing the DNA was transferred to a sterile 1.5 ml tube and stored in a freezer at a 20**°**C until amplification. Prior to sequencing, the PCR products were cleaned up using USB® ExoSAP-IT® PCR Product Cleanup. In brief, 5 μl of post-PCR reaction product was mixed with 2 μl Exo-SAP-IT reagent. This mix was then incubated at 37°C for 15 min, resulting in the degradation of any remaining primers and nucleotides. The samples were then incubated further at 80°C for 15 min to inactivate the ExoSAP-IT reagent.

### 2.5 Data preparation and statistical analysis

Cortisol concentrations were log10-transformed prior to analysis. Longitudinal cortisol across sampling timepoints (T1 to T6) was analyzed using a linear mixed-effects model with fixed effects of group, time (modeled as a categorical factor), sex, age, and the Group × Time interaction. A random intercept for participant ID was included to account for between-subject heterogeneity and non-independence of repeated measures within individuals. Within-subject residual covariance was modeled using a compound symmetry (CS) structure. Models were estimated using restricted maximum likelihood (REML), and Type III tests were used to evaluate fixed effects. The mixed-effects model used all available cortisol observations and accommodated missing repeated outcomes via likelihood-based estimation under a missing-at-random assumption. Participants contributed all available timepoint observations; participants with cortisol missing at all timepoints did not contribute outcome information to the longitudinal cortisol model. Because age and sex were included as covariates, cases with missing age or sex were excluded from the adjusted model.

CAR was summarized using area under the curve with respect to ground (AUCg) and with respect to increase (AUCi) computed from raw cortisol values across T2 to T6 (0, 15, 30, 45, and 60 min post-awakening) using the trapezoidal method; AUCi was calculated as AUCg minus the baseline (T2) area across the 0 to 60 min interval. Group differences in AUCg and AUCi were examined using univariate general linear models (UNIANOVA) with group and sex as categorical factors and age and T1 cortisol as continuous covariates (Type III sums of squares). AUCg and AUCi were calculated using complete-case CAR data (T2 to T6); participants missing any CAR sample did not contribute to these analyses, and cases with missing covariates were excluded by the model-fitting procedure.

To explore associations between depressive symptom severity and CAR summary measures, separate multiple linear regression models were conducted with AUCg and AUCi as dependent variables. In each regression model, BDI score was included as the predictor of interest, and age and sex were included as covariates. Analyses used listwise deletion such that only participants with non-missing values for the dependent variable and all predictors were included.

5-HTTLPR has been described as functionally triallelic. The L_G_ allele, which is the L allele with a common G substitution (rs25531, A/G), yielded lower mRNA levels than the L allele with an A substitution (L_A_). Expression from the S and L_G_ alleles is nearly equivalent [24]. The results were categorised based on the triallelic model (high versus low expressive). Only the MDD group provided buccal cell DNA for genotyping.

The cortisol sampling protocol required adequate saliva secretion and consisted of five consecutive morning samples collected at 15 min intervals (T2 to T6). Some samples could not be assayed due to insufficient biological material, resulting in missing cortisol values. Longitudinal analyses used linear mixed-effects models that incorporate all available observations and accommodate missing repeated measures via likelihood-based estimation (under a missing-at-random assumption). The distribution and frequency of missing cortisol samples by timepoint and group (including genotype and HC stratification) are reported in Supplemental Table 2. The data preparation, analysis, and visualisations were performed using the statistical software SPSS Statistics 27 and RStudio 2023.12.

## 3 Results

**Table 1:** Sample characteristics.

Variable	MDD SS	MDD LS	MDD LL	HC
Age, M years (SD)	39 (13.9)	39 (12.5)	34 (11.2)	41 (13.1)
ISCED level, M (SD)	6.0 (.9)	5.8 (1.3)	6.0 (.9)	5.9 (.9)
Sex, n females (%)	19 (79)	39 (61)	13 (65)	40 (68)
BDI-II, M (SD)	14.4 (9.6)	14.7 (10.5)	15.7 (12.1)	1.7 (3.1)
Number of previous MDE, M (SD)	3.1 (1.4)	3.1 (1.3)	2.9 (1.3)	0 (.0)
On psychotropic medication, n (%)	3 (12)	24 (37)	6 (30)	0 (0)
Total	24	64	20	59

*Note*. ISCED, International Standard Classification of Education; BDI-II, Beck Depression Inventory; MDE, major depressive episode; MDD SS, previous major depression with two short 5-HTTLPR alleles; MDD LS, previous major depression with one short 5-HTTLPR allele; MDD LL, previous major depression with no short 5-HTTLPR allele; HC, healthy controls (not genotyped).

## 4 Cortisol analyses in MDD genogroups versus healthy controls

### 4.1 Linear mixed model (LMM)

**Fig. 1:**
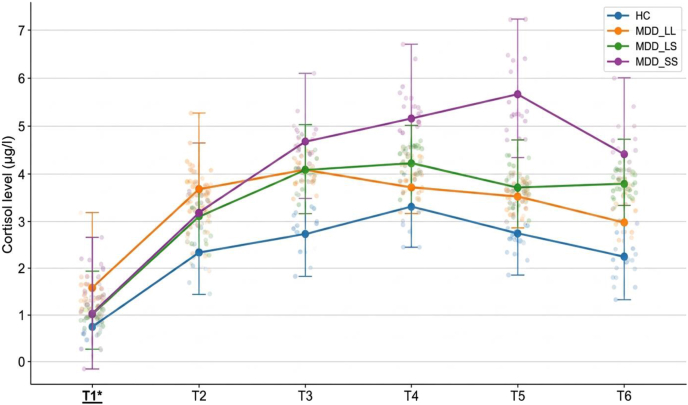
Cortisol trajectory of MDD Genogroups and Healthy Controls Note. T1* represents the evening (night) cortisol sample. T2–T6 represent the five consecutive post-awakening samples at 15-min intervals. Whiskers indicate 95% confidence intervals.

A linear mixed-effects model (CS residual covariance, random intercept for participant) was fitted with log10-transformed cortisol (lcort) as the dependent variable and fixed effects of group, time, sex, age, and the Group × Time interaction. There was a significant main effect of time, F(5, 534.49) = 30.85, p < .001, ηp^2^ = .224. The main effect of group was not statistically significant, F(3, 107.47) = 2.44, p = .068. The Group × Time interaction was not statistically significant, F(15, 534.11) = 1.40, p = .143, indicating no evidence that the pattern of change over time differed across groups. Sex was not significantly associated with lcort, F(1, 105.58) = .56, p = .457, and age was not significant, F(1, 104.98) = 2.05, p = .155.(Fig. 1).

### 4.2 Area under the curve with respect to increase (AUCi)

A univariate general linear model was used to examine group differences in AUCi (raw cortisol), computed from T2 to T6 (0–60 min post-awakening), while adjusting for age, sex, and T1 cortisol. The overall model was not statistically significant, F(6, 95) = 1.44, p = .209, R^2^ = .083 (adjusted R^2^ = .025). There was no significant main effect of group, F(3, 95) = 1.01, p = .392, and T1 cortisol was not a significant predictor of AUCi, F(1, 95) = 1.45, p = .231. Age was not significantly associated with AUCi, F(1, 95) = 2.94, p = .090, and sex was not significant, F(1, 95) = .33, p = .566.

### 4.3 Area under the curve with respect to ground (AUCg)

**Fig. 2:**
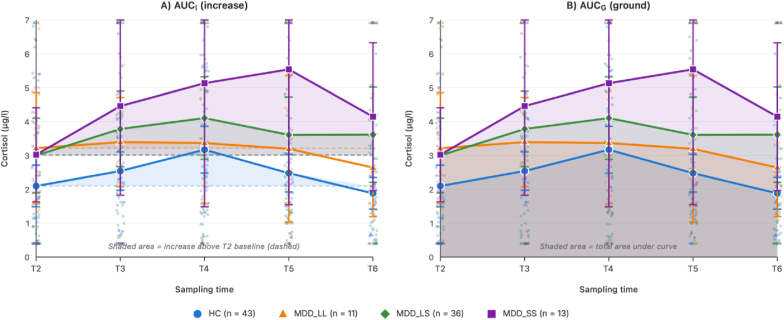
AUC analysis of morning cortisol by 5-HTTLPR genotype Note. A) AUCi: area under the cortisol awakening curve with respect to increase from baseline (T2 mean, dashed lines). B) AUCg: total area under the curve with respect to ground (zero). Five morning samples: T2 = awakening, T3 = +15 min, T4 = +30 min, T5 = +45 min, T6 = +60 min. Whiskers indicate 95% confidence intervals. Individual data points shown with jitter.

A univariate general linear model was used to examine group differences in post-awakening cortisol output (AUCg; raw cortisol), computed from T2 to T6 (0–60 min post-awakening), while adjusting for age, sex, and T1 cortisol. The overall model was statistically significant, F(6, 95) = 20.35, p < .001, R^2^ = .562 (adjusted R^2^ = .535). T1 cortisol was a significant predictor of AUCg, F(1, 95) = 104.60, p < .001, ηp^2^ = .524. There was a significant main effect of group, F(3, 95) = 2.83, p = .042, ηp^2^ = .082. Age was not significantly associated with AUCg, F(1, 95) = .26, p = .612, and sex was not significant, F(1, 95) = .18, p = .677 (Fig. 2).

### 4.4 Exploring AUCi and AUCg, and depression symptoms

The overall regression model predicting AUCi from BDI, age, and sex was not statistically significant, F(3, 99) = 1.50, p = .219, R^2^ = .044 (adjusted R^2^ = .015). BDI was not significantly associated with AUCi, B = .91, SE = 1.26, β = .072, t(99) = .72, p = .471, 95% CI [−1.58, 3.40]. Age was a significant positive predictor of AUCi, B = 2.11, SE = 1.04, β = .206, t(99) = 2.03, p = .045, 95% CI [.04, 4.18]. Sex was not significant, B = 23.91, SE = 29.23, β = .082, t(99) = .82, p = .415, 95% CI [−34.09, 81.91]. The overall regression model predicting AUCg from BDI, age, and sex was not statistically significant, F(3, 99) = .69, p = .563, R^2^ = .020 (adjusted R^2^ = −.009). BDI was not significantly associated with AUCg, B = 1.54, SE = 1.56, β = .100, t(99) = .99, p = .324, 95% CI [−1.54, 4.63]. Age was not significant, B = .56, SE = 1.29, β = .045, t(99) = .44, p = .664, 95% CI [−2.00, 3.13], and sex was not significant, B = 36.11, SE = 36.24, β = .101, t(99) = 1.00, p = .321, 95% CI [−35.79, 108.02].

## 5 Discussion

We investigated whether 5-HTTLPR genotype groups within the depression-history sample differed from a healthy control group in morning cortisol dynamics. Across analyses, cortisol showed a clear change over time from T1* to T6 in the linear mixed-effects model, consistent with a pronounced time effect across the sampling window. However, the overall pattern of change over time did not differ significantly between genotype groups within the depression-history sample (no Group × Time interaction), indicating limited evidence that genotype modified the shape of the cortisol trajectory across the repeated samples. When cortisol was summarized as post-awakening indices, results differed for overall output versus reactivity when comparing the depression-history genotype groups with healthy controls. AUCg showed a significant group effect after adjusting for the evening sample and covariates, and evening cortisol was a strong predictor of AUCg, suggesting that baseline evening cortisol level accounted for substantial variance in subsequent morning output. In contrast, AUCi did not show significant group differences under the same adjustment, indicating that group effects were more apparent for overall cortisol exposure than for the awakening-related increase component. Finally, exploratory regressions examining residual depressive symptoms did not provide evidence that symptom severity was associated with either AUCg or AUCi, suggesting that the observed cortisol differences were not explained by residual symptom burden in this sample. While prior meta-analytic work has suggested that short-allele carriers may show higher cortisol responses to psychosocial stress [16,25], our findings provide only partial and measure-dependent support for genotype-related differences in post-awakening cortisol in a previously depressed sample. Our analyses provided limited evidence that genotype groups differed in the shape of the cortisol trajectory across the repeated morning samples, which suggests that any genotype-related differences in this dataset were not strongly expressed as differential change over time.

Stress reactivity is not uniformly heightened in depression. Indeed, the broader literature documents substantial heterogeneity, including evidence for lower or blunted physiological responses among individuals with remitted depression and across depressive phenotypes [[26], [27], [28], [29]]. Consequently, our results support a cautious interpretation where group-related differences, if present, may not necessarily manifest as a differential time course in the first hour after awakening. A more nuanced interpretation is also consistent with the literature on the direction of CAR alterations and what they may signify. A larger CAR has been linked in some work to better coping with daily stressors, resilience, and lower worry [[30], [31], [32]], while lower or blunted CAR patterns have been observed in the context of chronic stress exposure [33] and in subclinical depressive presentations [34]. These findings emphasize that CAR variation may reflect multiple underlying processes, including anticipatory processes related to the upcoming day and longer-term stress context, rather than a single “hyperreactive” profile in all individuals with depression history.

Our exploratory results are consistent with the possibility that 5-HTTLPR-related differences may be detectable in some aspects of HPA-axis output under specific modeling choices (e.g., accounting for evening cortisol), while not necessarily manifesting as symptom-linked cortisol differences. It should be noted that all MDD participants had a history of depression and were not in a current depressive episode at the time of assessment, though many reported residual symptoms on the BDI-II. This pattern is compatible with a diathesis-stress perspective and with the original Caspi et al. [35] proposition that genetic variation may not exert uniform main effects but instead shapes how individuals respond to environmental challenge. Group differences in overall cortisol output (AUCg) when adjusting for evening/night cortisol are consistent with the idea that genetic influences in the depression-history sample may emerge more clearly when the physiological context is specified, and baseline-related variance is accounted for. Taken together, these findings align with later literature showing that replication of the Caspi interaction has been inconsistent [[36], [37], [38], [39]]. Any genotype-by-stress association likely depends on features of stress measurement such as chronicity and timing relative to symptom assessment [40].

Our study has limitations that should be considered alongside its strengths. First, depressive symptoms were assessed using the BDI-II at a single time point, and “residual symptoms” were therefore broadly operationalized; we did not have longitudinal symptom data, detailed information on episode duration or severity, or data on treatment response and timing since the last episode. We also could not quantify lifetime antidepressant exposure or dosage, which may be relevant given potential medication effects on HPA-axis functioning [41]. In addition, CAR was assessed on a single day. Single-day CAR estimates are associated with substantially reduced reliability and increased susceptibility to transient state-related influences compared with multi-day protocols [42]. Objective verification of awakening and sampling times was not available, further limiting confidence in timing accuracy. The genotyped subgroup sizes were modest, which may limit generalizability and reduce power to detect smaller effects. Furthermore, epigenetic moderation of 5-HTTLPR associations with cortisol has been reported [43], but methylation data were not available in the present study. Critically, because healthy controls were not genotyped, it is not possible to disentangle whether the observed AUCg group effect reflects genotype-specific influences on cortisol, a general effect of depression history, or some interaction between the two. Despite these constraints, the study has several notable strengths. It combines genotyping with intensive endocrine sampling and structured clinical assessment, yielding more than one thousand individual cortisol observations. Analytically, we used complementary approaches that align with contemporary CAR methodology, including mixed-effects modeling of repeated measures and AUC-based summary indices that distinguish total post-awakening output (AUCg) from baseline-relative change [44]. The observed divergence between AUCi and AUCg is consistent with current consensus guidance that the term “CAR” should refer specifically to the dynamic post-awakening increase rather than total post-awakening output [42], and this distinction is important for interpretation. Taken together, the current work provides preliminary but methodologically informed evidence relevant to how 5-HTTLPR variation and depression history may relate to post-awakening cortisol measures, while underscoring the need for larger, pre-registered studies with multi-day CAR assessment and objective adherence verification.

## 6 Conclusion

Cortisol changed across the sampling window, but genotype groups did not differ in the overall cortisol time course. Group differences were more evident for overall post-awakening output whereas baseline-relative increase showed no corresponding group differences. BDI-II was not associated with AUC-based CAR indices. Given the exploratory nature of these analyses, the modest sample sizes, and the absence of genotyping in controls, more definitive conclusions will require larger, pre-registered studies with multi-day CAR assessment, objective adherence verification, and genotyping across all participant groups.

## Ethical considerations

The research project received approval from the Regional Committee for Health Research for South-Eastern Norway (REK Sør-Øst 2014/21). All participants involved in the study provided written informed consent. The biobank used in this research was approved by REK Nord t6/2006.

## Declaration of generative AI and AI-assisted technologies in the manuscript preparation process

We used Sikt KI-chat, an AI-powered language support tool provided by Sikt (the Norwegian Agency for Shared Services in Education and Research), to check grammar and spelling in the manuscript. Following this linguistic review, the manuscript was sent to Scribendi for editing in accordance with APA 7th edition guidelines and for proofreading. These tools were used solely for language quality assurance; all scholarly content, interpretations, and conclusions are the authors’ own.

## Funding

This work was supported by the South-Eastern Norway Regional Health Authority 2015052 [to NIL], the Research Council of Norway 229135 [to NIL], and 175387/V50 [to NIL].

## CRediT authorship contribution statement

**R. Jonassen:** Conceptualization, Data curation, Formal analysis, Funding acquisition, Investigation, Methodology, Project administration, Resources, Visualization, Writing – original draft. **Ø. Øverli:** Data curation, Investigation, Methodology, Supervision, Writing – review & editing. **E. Hilland:** Conceptualization, Writing – review & editing. **H.L. Nilsen:** Data curation, Formal analysis, Investigation, Methodology, Writing – review & editing. **Q.Y. Esbensen:** Data curation, Formal analysis, Methodology, Writing – review & editing. **L. Lirussi:** Data curation, Formal analysis, Methodology, Writing – review & editing. **H. Heiberg:** Data curation, Formal analysis, Investigation, Writing – original draft. **K.H. Rønning:** Data curation, Formal analysis, Writing – review & editing. **M. Gorissen:** Data curation, Formal analysis, Methodology, Supervision, Writing – review & editing. **B. Kraft:** Conceptualization, Supervision, Writing – review & editing. **R. Bø:** Conceptualization, Data curation, Writing – review & editing. **C.J. Harmer:** Conceptualization, Funding acquisition, Supervision, Writing – review & editing. **N.I. Landrø:** Conceptualization, Funding acquisition, Writing – review & editing.

## Declaration of competing interest

The authors declare that they have no known competing financial interests or personal relationships that could have appeared to influence the work reported in this paper.
