# Supplementary material for: Serotonin transporter polymorphism (5-HTTLPR) is associated with post-awakening cortisol measures in previous depression
Source: Compr Psychoneuroendocrinol. 2026 Jul 22;27:100363. doi: 10.1016/j.cpnec.2026.100363 (PMC13425797; doi:10.1016/j.cpnec.2026.100363)
Supplement: Multimedia component 1 [file mmc1.docx]

**Supplementary Material**

**Supplemental Table 1**

Number of Earlier Episodes * 5-HTTLPR and HC Crosstabulation

| Number of earlier episodes | MDD SS | MDD LS | MDD LL | HC |  |
| --- | --- | --- | --- | --- | --- |
| 0 | 0 | 0 | 0 | 59 | 59 |
| 1 | 3 | 6 | 2 | 0 | 11 |
| 2 | 6 | 19 | 7 | 0 | 32 |
| 3 | 6 | 13 | 6 | 0 | 25 |
| 4 | 2 | 10 | 0 | 0 | 12 |
| 5 ≥ | 7 | 16 | 5 | 0 | 28 |
| Total | 24 | 64 | 20 | 59 | 167 |

Note. Frequencies of MDD episodes for MDD genogroups and HC as defined by module A of MINI 6.0. Patients with more than five episodes are collapsed into one group (≥ 5). A total of 11 patients experienced only one episode of MDD.

**Supplemental Table 2**

| *Number of Missing Cortisol Samples * 5-HTTLPR and HC Crosstabulation* | | | | | | |
| --- | --- | --- | --- | --- | --- | --- |
|  | | MDD SS | MDD LS | MDD LL | HC | Sum |
| Cortisol sample (T_n)_ | T1 | 6 | 20 | 6 | 12 | 44 |
|  | T2 | 8 | 20 | 5 | 12 | 45 |
|  | T3 | 7 | 20 | 5 | 12 | 44 |
|  | T4 | 10 | 28 | 9 | 13 | 60 |
|  | T5 | 10 | 28 | 9 | 12 | 59 |
|  | T6 | 10 | 27 | 9 | 14 | 60 |
| Total | | 51 | 143 | 49 | 75 | 312 |

Note. Number of missing values at different time points of MDD and HC groups.

*Model Diagnostics and Sensitivity Analyses*

Levene’s test indicated that the assumption of homogeneity of error variances was not violated for either outcome. For AUCi, Levene’s test was not significant, F(7, 94) = 0.83, p = .565. For AUCg, Levene’s test was also not significant, F(7, 94) = 1.28, p = .269. AUC analyses were conducted on the complete-case post-awakening sample (T2–T6) with complete covariate data (N = 102). Standardized residuals from the mixed-effects model on log10-transformed cortisol were inspected for extreme values. Across the 1002 person-by-time observations in the dataset, 15 observations (1.5% of all observations; 2.2% of non-missing residuals) exceeded |z| > 3. A sensitivity analysis excluding cases flagged for extreme residuals produced the same pattern of findings: time remained significant, F(5, 509.59) = 32.82, p < .001, and neither the main effect of group, F(3, 111.48) = 2.55, p = .059, nor the Group × Time interaction, F(15, 509.05) = 1.30, p = .195, reached statistical significance. An AR(1) residual covariance structure was also evaluated for the LMM; however, the random-intercept variance was estimated as zero and flagged as redundant, indicating an overparameterized covariance structure and yielding an inadmissible Hessian. Therefore, inference is reported from the CS model with a random intercept, which yielded admissible variance component estimates.
